# Quercetin as a Dietary Supplementary Flavonoid Alleviates the Oxidative Stress Induced by Lead Toxicity in Male Wistar Rats

**DOI:** 10.3390/nu15081888

**Published:** 2023-04-14

**Authors:** Mohammed Al-Zharani, Mohammed Mubarak, Hassan Ahmed Rudayni, Amin A. Al-Doaiss, Mahmoud M. Abd-Elwahab, Mohammed S. Al-Eissa

**Affiliations:** 1 Department of Biology, College of Science, Imam Mohammad Ibn Saud Islamic University (IMSIU), Riyadh 11623, Saudi Arabia; 2 Department of Biology, College of Science, King Khalid University, Abha 62529, Saudi Arabia; 3 Department of Mathematics and Statistics, College of Science, Imam Mohammad Ibn Saud Islamic University (IMSIU), Riyadh 11623, Saudi Arabia

**Keywords:** quercetin, lead, toxicity, antioxidant, biochemical profile

## Abstract

Quercetin is a naturally existing plant pigment belonging to the flavonoid group; it is contained in a wide range of vegetables and fruits. The accumulated evidence points to the potential uses of quercetin in protection of some disease conditions. Lead is one of the highly toxicant heavy metals that are widely spread in the environment and implicated in a wide spectrum of industries. No previous study has been reported to evaluate the effect of quercetin on lead toxicity. Therefore, the present study was conducted to elucidate some aspects of quercetin bioactivities in regard to its ability to combat the oxidative stress induced by lead toxicity. For this purpose, a total of sixty male Wistar rats were randomly and equally divided into three groups of 20 animals each; untreated control animals (group 1), lead-exposed animals (group 2; exposed to lead daily by oral gavage at the dose of 80 mg/Kg b.w.), and group 3 of animals, which were exposed to lead and daily received quercetin (10 h gap time between lead exposure and the receiving of quercetin) by oral gavage at the dose of 350 mg/Kg b.w. The experiment period was 8 weeks. All the assayed hematological and biochemical parameters of animals exposed to lead were significantly altered compared with the untreated control levels. Animals exposed to lead (group 2) exhibited significant decrements of the erythrocytic and total leucocytic counts, hemoglobin concentration, packed cell volume percent, total proteins, albumin and globulin. These animals also disclosed significantly decreased levels of antioxidant markers including total thiols, catalase and glutathione. On the other hand, these animals demonstrated significant increments in the levels of bilirubin, urea, creatinine, BUN, serum enzymes, H_2_O_2_ and MDA. Animals exposed to lead and given quercetin (group 3) exhibited improvement of these parameters, which were brought back at varying degrees toward the untreated control levels. Basing on the improvements of the assayed hematological and biochemical parameters, it was concluded that quercetin as a dietary supplement can act efficiently as an antioxidant to counteract the oxidative stress induced by lead toxicity and to maintain the oxidant antioxidant balance.

## 1. Introduction

The name quercetin is derived from the Latin word “Quercetum” (Oak Forest). Quercetin (3, 3, 4, 5, 7-pentahydroxyflavone) is a naturally existing ubiquitous plant flavonoid that belongs to the class flavanols. It is a plant secondary metabolite and chemically represents the aglycone form of flavonoid glycosides [1,2]. The term flavonoid comes from the name “flavus” (meaning yellow), and flavonoids are phenolic substances constituting a group of plant pigments. Quercetin as a plant flavonoid (phytofalvonoid) is contained in a wide range of vegetables and fruits including berries, cherries, herbs, spices, onions, tomatoes, apples, citrus fruits, red grapes, broccoli, peppers and leafy vegetables as well as plant-sourced beverages such as coffee and green tea [3]. All these food (edible) sources contain considerable concentrations of this nutraceutical bioflavonoid (flavonoid-rich vegetables and fruits), but the highest content (the richest source) is that found in onions [4]. Considering plant flavonoids, quercetin is the most prevalent and constitutes the largest proportion of flavonoids contained in the various food sources. Moreover, among the well-recognized flavonoids in fruits and vegetables, flavonols, especially quercetin, are the most prominent and the most-consumed in regular diets [5]. According to previously reported two different extraction protocols of quercetin, namely the ultrasound-assisted glycerol extraction (UAGE) and microwave-assisted extraction (MAE), and by the aid of HLPC-UV as a method of analysis, the extraction yields were 16.55 and 27.20 mg/1g red onion (dry weight), respectively [6].

Quercetin is not synthesized in the human or animal body [2]; hence, it should be obtained from one of its food sources or received as a dietary supplement. Some dietary constituents, such as bromelain (from pineapple) enhances absorption of quercetin and thus increases the benefits of even minute amounts of quercetin. Flavonoids in general are categorized as a group of biomolecules that have the capability to protect cells against the damaging effects [6]. As a polyphenolic substance of plant origin, a considerable number of animal, human and cell culture studies elucidated some quercetin bioactivities including its anti-inflammatory and anticancer properties [2,7,8,9]. There is accumulating evidence that points to the quercetin’s (a bioactive flavonoid) potential prophylactic and therapeutic uses in the protection of some disease conditions involving cardiovascular diseases, neurodegenerative disorders, diabetes, osteoporosis, arthritis, gastropathies and nephropathies as well as tumors (anticancer) [10,11,12]. The potent pharmacological properties of quercetin and its derivatives enable their wide spectrum implications as anti-viral, anti-allergy, anti-ulcer, anti-diabetic, anti-infective, antihypertensive, anti-obesity, antihypercholesterolemic, vasodilator, antiaging, antiplatelet, anti-arthritis and anti-atherosclerotic [13,14,15,16,17,18,19,20]. Moreover, quercetin was considered as a valid preventive and therapeutic agent of SARS-CoV-2 infection, especially in combination with zinc and vitamins C, D, and E [21]. Quercetin performs its preventive roles through modifying the mechanistic pathways of some biochemical processes [3,5]. In this regard, quercetin was found to prevent morphological alterations of RBCs (formation of acanthocytes) induced by H_2_O_2_ by counteracting the resultant oxidative damage [22]. Additionally, quercetin reduces Ca^2+^-induced hemolysis through inhibiting the main cation pathways including Ca^2+^-activated K^+^ channel [23]. Quercetin was also found to maintain RBCs’ deformability as evidenced by its protective effect of an erythrocyte membrane against the cigarette tar extract-induced membrane damage via scavenging reactive oxygen species (ROS) [24]. The protective effects of quercetin were found to extend to peripheral blood lymphocytes, since it helps protect against the nicotine-induced cellular and DNA damage through suppressing the process of lipid peroxidation [25]. The neuroprotective role of quercetin is mainly related to the suppression of the oxidative damage which could be triggered by hypoxia and ischemia [26]. The anti-cancer property of quercetin has been ascribed to its potential property to inhibit the proliferation of cancer cells through induction of apoptosis and cell cycle arrest as well as suppression of migration and invasion activities of cancer cells [27]. The preventive and therapeutic effect of quercetin in cases of osteoarthritis is presumably related to the inhibition of IL-1β and TNF-α production [28]. One of the antiviral mechanisms of quercetin is exemplified by its anti-HCV activity through inhibiting HCV-induced ROS and reactive nitrogen species (RNS) generation as well as suppressing lipoperoxidation [29]. Additionally, quercetin can inhibit virus entry such as the situation with influenza A virus [30].

As regard to its safety, quercetin is characterized by its GRAS (Generally Recognized As Safe) status, and no side effects have yet been documented in animal or human investigations. Because of the aforementioned pharmacological properties and bioactivities, quercetin has been focused on in a considerable number of human studies [31,32,33,34,35].

Lead is one of the highly toxicant heavy metals that are widely spread in the environment and implicated in a wide spectrum of industries [36,37,38]. By nature, lead is not vulnerable to biodegradation and is steadily accumulated, and this significantly increases the possible environmental hazards [39,40]. The most common source of toxicity to human is the occupational exposure to lead compounds [41,42,43]. Additionally, ingestion of contaminated food and water is usually considered in cases of lead poisoning. After this accidental ingestion, lead is rapidly absorbed to reach blood circulation and eventually lead is deposited in certain tissues [44]. However, as a heavy metal with highly toxic properties, lead can affect all types of tissues in animals and human [45,46].

Chronic exposure to lead has been incriminated in the causation of remarkable histopathological changes as a sequel to the prolonged lead-induced toxicological effects [47]. Hepatic and renal tissues are the remarkably affected as evidenced by the biochemical, histopathological and histochemical changes reported in the previous studies on lead toxicity [48,49,50,51,52].

To the best of our knowledge, no previous investigation has been reported concerning the role of quercetin to counteract the oxidative damage induced by lead toxicity. Therefore, the present study was undertaken to elucidate some aspects of quercetin bioactivities as regard to its potential property to combat the oxidative stress induced by lead toxicity.

## 2. Materials and Methods

### 2.1. Experimental Animals

A total of 60 adult male Wistar rats, aged 4 months and weighing between 160–200 g, were used in the present work. The animals were housed and maintained under the standard laboratory conditions (12 h dark–light cycle, ambient temperature 23 ± 1 °C, relative humidity of 35% to 70%). All the guides and rules to the care and use of experimental laboratory animals stated by the official “Research Ethics Committee of Imam Mohammad Ibn Saud University, Saudi Arabia” (LAB-animals-022-0139) were strictly applied.

### 2.2. Lead and Quercetin

Lead was used as lead acetate trihydrate (Pb (CH_3_COO)_2_ ×3 H_2_O) (Sigma-Aldrich, Darmstadt, Germany).

Quercetin (3, 3′, 4′, 5, 7-pentahydroxyflavone) was used as “quercetin Phytosome” (*Sophora japonica* extract/phospholipid complex) (Thorne Research Inc., New York, NY, USA).

The other ingredients contained in the quercetin product include leucine, microcrystalline cellulose, hypromellose and silicon dioxide. According to the manufacturer, this formula of quercetin product is characterized by the binding of quercetin to sunflower-sourced phospholipids to enhance quercetin absorption. The phytosome complexes are created by a patented process that binds a botanical extract to phospholipids; thus, these complexes carrying the botanical extract (quercetin) can easily cross the intestinal barrier to reach blood circulation.

### 2.3. Experimental Design

Animals were acclimatized for one week prior to experiment, and then randomly allotted into three equal groups, of 20 animals each, designated groups 1, 2 and 3. Group 1 served as untreated control, i.e., animals in this group were not exposed to lead and did not receive quercetin. Animals in group 2 were exposed daily to lead acetate trihydrate dissolved in water at the dose of 80 mg/Kg b.w. by an oral gavage in a volume of 1 mL/kg b.w. Rats in group 3 were exposed to lead, then given quercetin phytosome daily (10 h gap time between lead exposure and the receiving of quercetin) at the dose of 350 mg/Kg b.w. by an oral gavage in a volume of 1 mL/kg b.w.

Experiment period was 8 weeks; feed (dry ration) and drinking water were supplied ad libitum throughout the whole period.

All experimental animals were observed for feed consumption and water intake, as well as behavioral activity and clinical signs.

### 2.4. Hematological and Biochemical Assays

At the end of experiment, blood samples were collected from animals in all groups. The blood samples collected with an anticoagulant (EDTA) were used to assess the various hematological parameters, including RBCs and total WBCs counts, and also the other erythrocytic indices involving hemoglobin (Hb) concentration and packed cell volume (PCV)%.

Serum separated from the coagulated blood samples was utilized to estimate the various biochemical parameters encompassing total proteins, albumin, globulin, creatinine, urea, blood urea nitrogen (BUN), bilirubin, alanine aminotransferase (ALT), aspartate aminotransferase (AST), alkaline phosphatase (ALP), total thiols, glutathione (GSH), catalase, hydrogen peroxide (H_2_O_2_) and malondialdehyde (MDA).

The convenient hemocytometer was used to estimate the erythrocytic and total leucocytic counts and a micro-hematocrit method was employed to measure the packed cell volume (PCV)%. Hemoglobin (Hb) concentration was measured by Cyanmet-hemoglobin methods previously described [53,54].

Level of total thiols was assessed by a total thiol colorimetric assay kit (Cell Biolabs Inc., San Diego, CA, USA). Glutathione (GSH) level was determined by a reduced glutathione colorimetric assay kit (ElabScience, Houston, TX, USA). Catalase level was estimated by using a catalase activity colorimetric assay kit (BioVision, Abcam, Cambridge, UK). H_2_O_2_ level was determined with the aid of a colorimetric assay kit (Elabscience, Houston, TX, USA). Measurement of MDA was done by a colorimetric assay kit for MDA (Elabscience, Houston, TX, USA).

The levels of serum enzymes ALT, AST and ALP were determined by using diagnostic kits (BioMerieux, Marcy-IEtoile, France). Urea level was estimated by a colorimetric assay kit (BioVision, Biovision Incorporated, Cambridge, UK). BUN level was assessed by a colorimetric detection kit (ThermoFisher Scientific, Waltham, MA, USA). Other parameters of the biochemical profile, including total proteins, albumin, globulin, creatinine and bilirubin were determined using colorimetric diagnostic kits (Interchim Diagnostics Biochemistry Kits, Montlucon, France).

### 2.5. Statistical Analysis

The obtained data from all animals were presented as means ± S.D., and then analyzed using a T-student test by applying a statistical analysis SPSS software (SPSS Inc., Chicago, IL, USA). *P*-values less than 0.05 (*p* ˂ 0.05) were considered statistically significant.

## 3. Results

Rats in group 2, which were exposed to lead and did not receive quercetin, exhibited decreased hematological parameters. The decrements of Hb concentration and PCV% were obviously significant. Group 3 animals, who were exposed to lead and concomitantly received quercetin, disclosed an improvement of these parameters toward the control levels.

Table 1 shows the hematological parameters in rats exposed to lead, and rats exposed to lead and given quercetin, compared to the control untreated rats.

**Figure 1:**
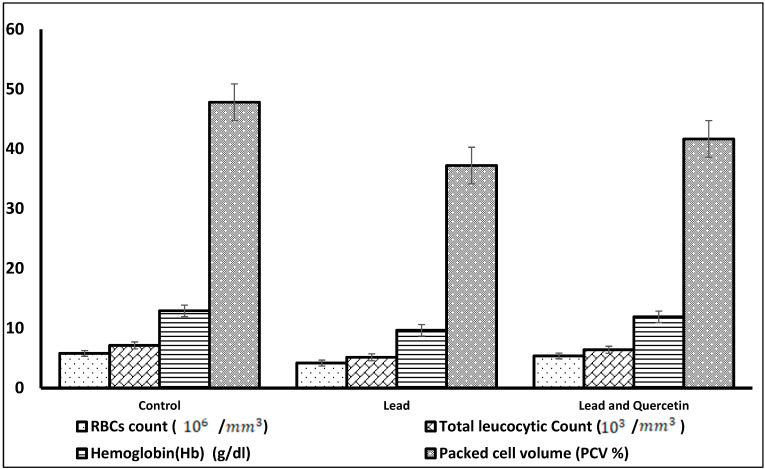
Hematological parameters in rats exposed to lead, and rats exposed to lead and given quercetin phytosome, compared to the untreated control rats.

Figure 1 shows a graphical representation of the hematological parameters in rats exposed to lead, and rats exposed to lead and given quercetin, compared to the untreated control rats.

Regarding the biochemical parameters, levels of total proteins, albumin and globulin were decreased in lead-exposed animals that did not receive quercetin (group 2) in relation to control levels. Rats in this group demonstrated significantly increased levels of urea, creatinine, BUN and bilirubin. The increments of urea and BUN levels were noticeable.

Total thiols, catalase and glutathione showed significantly decreased levels in rats exposed to lead and with no access to quercetin (group 2). MDA and H_2_O_2_ levels in animals of this group were significantly increased compared with the control levels. For all the assessed biochemical parameters, lead-exposed rats which received quercetin (group 3) demonstrated relative restore toward the control levels.

Table 2a–c shows the biochemical parameters in rats exposed to lead, and rats exposed to lead and given quercetin, compared to the control untreated rats.

**Figure 2:**
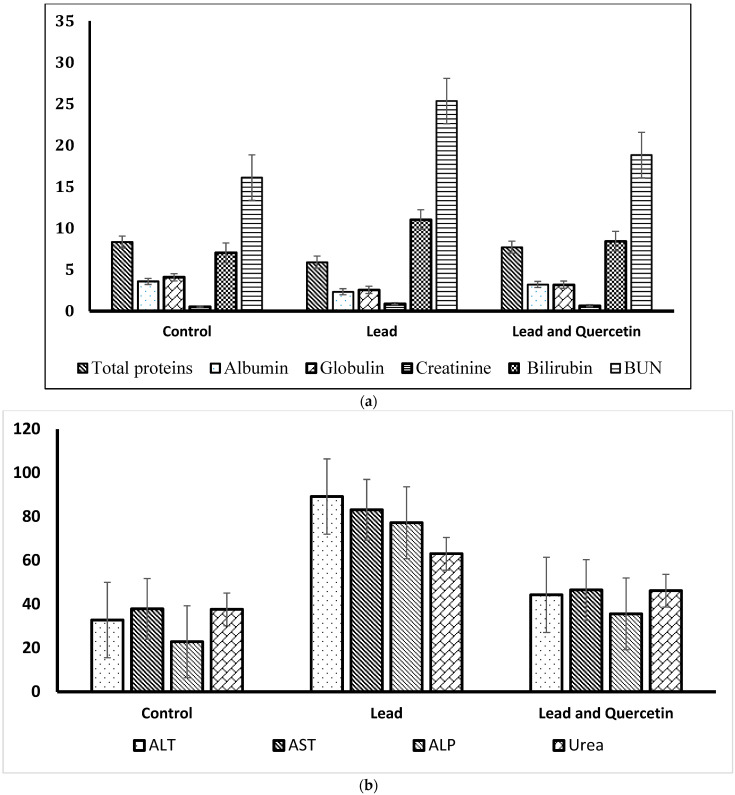

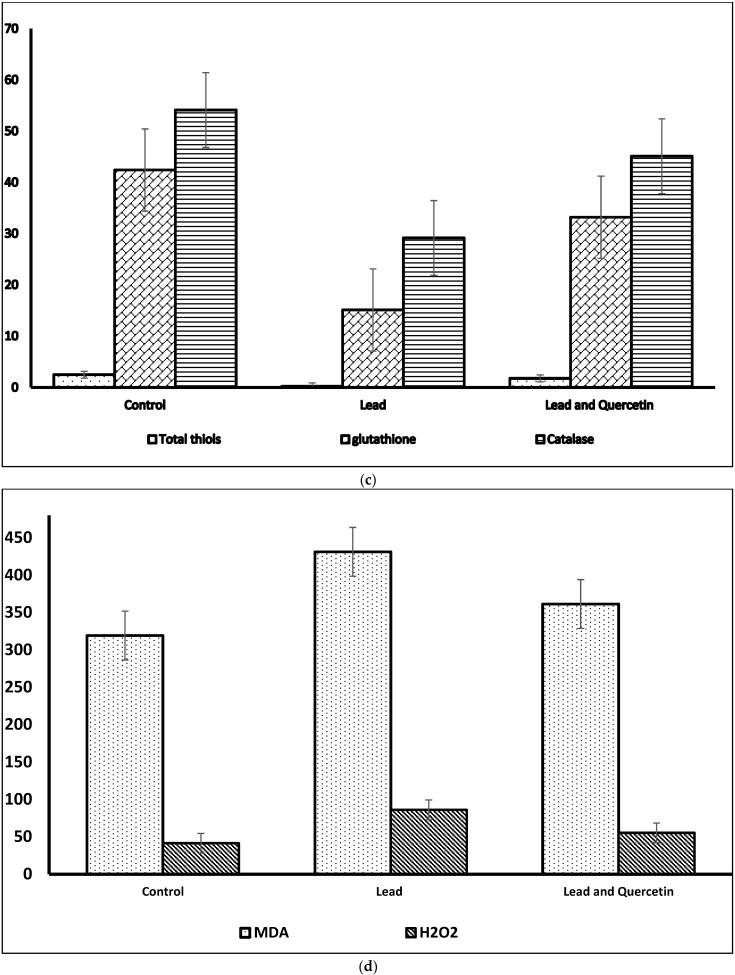
Biochemical parameters in rats exposed to lead, and rats exposed to lead and given quercetin phytosome, compared with the untreated control rats. (**a**) Levels of total proteins (g/dL), albumin (g/dL), globulin (g/dL), creatinine (mg/dL), BUN (mg/dL) and bilirubin(mg/dL). (**b**) Levels of alanine transferase (ALT) (IU/L), aspartate transferase (AST) (IU/L) alkaline phosphatase (ALP) (IU/L) and urea (mg/dL). (**c**) Levels of total thiols (mmol/L), glutathione (µg/mL) and catalase (IU/L). (**d**) Levels of malondialdehyde (MDA) (nmol/mL) and hydrogen peroxide (H_2_O_2_) (mmol/L).

Figure 2a–d shows graphical representations of the biochemical parameters in rats exposed to lead, and rats exposed to lead and given quercetin, compared to the untreated control rats.

## 4. Discussion

Quercetin was employed in the current research work as one of the highly consumed plant flavonoids. Polyphenols is the major chemical class which comprises flavonoid and non-flavonoid compounds [2]. Quercetin as a flavonoid compound that has five hydroxyl groups, and on glycosylation, these groups produce quercetin glycosides [1]. Bioactivity of quercetin is ascribed to its active phenolic hydroxyl groups [20].

The present study was conducted to test the potential of quercetin as a dietary supplement to alleviate the lead-induced toxicological effects. More specifically, the antioxidant property of quercetin was investigated in the presence of an oxidative stress induced by a highly toxicant heavy metal. The evaluation was mainly dependent on an assessment of the various hematological and biochemical parameters which are reliable indicators to the resultant alterations of tissues and organs.

Free radicals are constantly released in a controlled rate (physiological limits) from normal metabolic activities, are employed in some beneficial regular processes such as cell respiration and also beneficial for phagocyte activity against microbial infections [55]. Some free radicals, such as superoxide anions and nitric oxide (NO), function as mediators of certain physiological signaling pathways. Moreover, reactive oxygen species (ROS) and NO are integrated in regulation of O2 tension in gaseous change, vascular tone and production of erythropoietin [56].

Oxidative stress refers to the situation in the body when there is excessive uncontrolled generation of reactive oxygen species (ROS), which are unstable molecules of high reactivity, in response to various stimulants such as polluted air and water, pharmaceutical agents, toxins, microbial infections and heavy metals [26]. This situation is raised as a result of the imbalance between oxidation reactions and antioxidation activity. ROS, including free radicals, can provoke a variety of damaging effects that involve cell proteins and DNA as well as structural lipids.

Hydroxyl and superoxide radicals and the non-radical hydrogen peroxide are grouped under the reactive oxygen species (ROS). Free radicals are unstable molecules that tend to acquire electrons through reaction with the surrounding molecules or atoms to fill their outer shells. Therefore, in excess magnitude, free radicalsbecome highly reactive oxidants, i.e., oxidize other cellular biomolecules. Excess free radicals promote aging changes, and through induction of permanent tissue damage can lead to outstanding clinical disease conditions such as Alzheimer’s, cardiac disorders, nephropathies and cancer [21]. This state of exaggerated oxidation is referred to as oxidative stress and the resultant damage, which may involve membrane lipids, vital proteins and DNA, is known as oxidative damage [57,58]. Oxidation reactions in cell membranes provoke lipid peroxidation, which is a highly damaging process and may lead to cell death [59]. Free radicals through induction of permanent tissue damage may lead to outstanding clinical disease conditions such as Alzheimer’s, cardiac disorders, nephropathies and cancer [60].

Antioxidants, exogenous or endogenous, refer to the bioactive substances that delay, prevent, inhibit or eliminate the damaging oxidative effects of free radicals on vital cell biomolecules [38]. Endogenous antioxidants via electron donation can neutralize free radicals, and these endogenous molecules are crucial for the survival of living cells. The naturally existing endogenous antioxidant system is responsible for counteracting the damaging effects of excess free radicals and are actively engaged in the elimination of such deleterious radical molecules [50,61,62]. This endogenous system encompasses enzymes such as superoxide dismutase, catalase and glutathione peroxidase which in different pathways cooperate to neutralize and eradicate the excess of free radicals [63,64].

However, in some situations the magnitude of the oxidative stress overcomes the power of the endogenous antioxidant system, which suffers from depletion of its components under the effect of oxidation reactions. Thus, the balance (equilibrium) between the activity of oxidative molecules (free radicals) and the counteracting action of antioxidative molecules, which become oxidized nonfunctional molecules, is dramatically disturbed [50]. Oxidative stress induced by the highly toxic heavy metals is amongst the prevalent causes of such disrupted oxidation: antioxidation equilibrium. External antioxidants are mandatory in such cases to support function of the endogenous antioxidant system. External dietary supplements, which confer antioxidant activities, are preferred to readjust the oxidation: antioxidation balance. Prevention of lipid peroxidation is one of the main protective mechanisms through which antioxidant dietary supplements can support and enhance the endogenous antioxidative activity [65].

Thereafter, the external antioxidants, taken as a dietary supplement, act in a synergistic manner with the existing endogenous antioxidant molecules. The properly functioning synergistic mechanisms should inhibit the major free radicals-induced damaging effects, especially lipid peroxidation. Scavenging of free radicals, binding with pro-oxidative molecules and promotion of ROS reduction are main targets of these beneficial mechanisms [55,66].

The current results showed decreased hematological indices in rats exposed to lead; similar findings were reported previously in heavy metals toxicity [67]. The decreased erythrocytic and total leucocytic counts encountered in the present study may be attributed to the toxic effects of lead on the metabolic processes of blood cells, and a possible direct negative impact on hematopoiesis [68].

The current findings also showed significant alterations of biochemical parameters in lead intoxicated rats that did not receive quercetin. Biochemical alterations are usual consequences to cell and tissue damage. In case of lead toxicity, the induced damage is attributable to lead reactivity with tissue protein molecules as well as with subcellular nuclear structures and this damaging effect can initiate the generation of ROS [69].

Presently, levels of serum enzymes were significantly elevated in lead-exposed rats; the increments of serum ALT, AST and ALP reflect the lead-induced hepatic damage [70]. Lipid peroxidation, as a consequence of oxidative damage, renders lysosomal membranes more permeable and leakier and subsequently hepatocellular enzymes elevated in serum. Similarly, the increased biochemical parameters related to kidney functions, including urea, BUN and creatinine, indicate significant lead-induced renal damage.

Levels of total thiols, glutathione and catalase, which are components of the endogenous antioxidant system, were presently decreased in rats exposed to lead. These decrements reflect impaired capacity of the antioxidant system due to oxidation of the antioxidant enzymes and molecules by the released ROS. In this regard, thiols represent the largest part of the overall antioxidant pool [71]. Thiols act as electron acceptors; hence it can efficiently reduce and scavenge ROS [72]. Catalase is a crucial antioxidant enzyme; it decomposes hydrogen peroxide into oxygen and water and sustains the cellular redox homeostasis [73,74]. Catalase deficiency or dysfunction is related to the pathogenesis of some disease conditions such as cardiovascular diseases and neurological disorders [75]. Glutathione is the highest concentration non-protein thiol; it is synthesized in all cell types with the liver being the main source [76]. Beside its antioxidant property, glutathione plays a key role in the synthesis of protein and DNA, and maintenance of the cell redox potential [77]. Catalase and glutathione actively function to scavenge free radicals, and catalase provokes degradation of hydrogen peroxide and thus excludes the main factor responsible for lipid peroxidation [78]. Thus, depletion of catalase gives the opportunity for free radicals to provoke more intensive lipid peroxidation [79]. Thiols act mainly to scavenge singlet oxygen and hydroxyl radicals, and their depletion gives these radicals the chance to cause more damaging effects [80]. The presently increased level of MDA in rats exposed to lead is ascribed to lipid peroxidation of the cellular membranous structures. It has been shown that MDA is a reliable marker of lipid peroxidation associating oxidative damage [81]. Similarly, H_2_O_2_ was significantly elevated in these rats; H_2_O_2_ is one of ROS produced during the events of oxidative stress. It has a potent oxidizing property and it represents the key factor in Fenton’s reaction that generates the highly damaging hydroxyl radical (OH) [82].

The present results exhibited restored hematological and biochemical parameters in rats exposed to lead who then concomitantly received quercetin. These findings might indicate a certain capability of quercetin to ameliorate the resultant oxidative damage induced by lead. Flavonoids in general are known to have antioxidant bioactivity, and quercetin as a plant flavonoid has an antioxidant property, which is attributable to the phenolic hydroxyl groups and double bonds inserted in its chemical structure [83].

Quercetin can perform its antioxidant activity through multiple mechanistic pathways, including a direct mechanism to scavenge the excess ROS raised as a sequel to an oxidative damage [84]. This direct antioxidative action is strongly linked to the chemical structure of quercetin, which has two benzene rings (A and B rings) which fuse together to form a pyran ring. The benzene B ring has a characteristic affinity to react with and scavenge ROS and represents an active site for the antioxidant activity [9]. One of the antioxidant mechanisms of quercetin is its regulatory effect on glutathione level since it is an inducer of glutathione synthesis [85]. Additionally, quercetin contributes to combat ROS and to sustain the oxidative antioxidant balance through enhancing the expression of the endogenous antioxidant enzymes including superoxide dismutase, catalase and glutathione peroxidase. In this regard, it was found that quercetin markedly decreases ROS production in microglia cells in parallel with enhancing the antioxidant enzyme activity [86]. It is worth mentioning that quercetin, through reducing production of ROS induced by oxidative damage, helps protect mitochondria against the damage provoked by ultraviolet radiation [87]. The role of quercetin in the reparative process of DNA damage provoked by oxidative damage is still questionable despite the reported interaction between quercetin and DNA [86]. Beside its direct effect to eliminate free radicals, quercetin can protect cell membranes against lipid peroxidation through inhibiting oxidation of the lipoproteins and helps protect cells from the released peroxides [88,89]. In this regard, quercetin, when tested to protect fish oil from oxidation, was found to inhibit oxidation of polyunsaturated fatty acids, and consequently, aldehydes formation was compromised [90]. It has been demonstrated that quercetin reduces significantly MDA tissue level in kidney and liver, which undoubtedly implies significant inhibition of lipid peroxidation [91]. In addition, quercetin is able to inhibit oxidation enzymes such as xanthine oxidase and prevent oxidation of low-density lipoproteins (LDL) and accordingly blocks the events of atherosclerosis [88].

In the context of counteracting the oxidative damage, quercetin can function as a chelating agent to bind the pro-oxidant metal ions such as Cu^2+^ and Fe^2+^ [92]. Through binding Fe^2+^, quercetin inhibits Fe^2+^-mediated lipid peroxidation and minimizes the level of iron ions, which represent the fuel of the oxidation reactions and the subsequent oxidative damage [93]. In the presence of quercetin, Fe^2+^ ions become bound and inactive and thus fail to interact with hydrogen peroxide, i.e., blocking Fenton’s reaction [92]. In regard to the oxidative stress-induced apoptosis, long-term administration of quercetin in a diabetic rat experimental model was found to ameliorate the apoptotic changes via activation of the nuclear transcription factor-Nrf2 and reverse the impaired total antioxidant capacity (T-AOC) and the elevated MDA level [94]. Quercetin demonstrated an inhibitory effect on apoptotic pathways in the in vitro studies conducted to evaluate its counteracting effect on some apoptosis inducers [85,95].

Taking all in consideration, quercetin is regarded as an efficient versatile antioxidant through different pathways including direct scavenging of ROS, regulatory effect on glutathione synthesis, enhancing activity and expression of antioxidant enzymes, inhibitory effects on the oxidation enzymes, chelating action on pro-oxidant metal ions, prevention of lipid peroxidation and anti-apoptotic activity [85,88,96,97].

In view of these mechanisms, quercetin can significantly sustain the total antioxidant capacity and maintain the oxidant antioxidant balance, and thus alleviate greatly the damaging effects of the induced oxidative stress.

## 5. Conclusions

No previous study has been reported to evaluate the effect of quercetin on lead toxicity. Therefore, the present study was carried out to elucidate some aspects of quercetin bioactivities in regard to its ability to combat the oxidative stress induced by lead toxicity.

Presently, the levels of hematological parameters in rats given quercetin were brought back to approach the control levels. This suggested the ability of quercetin to remarkably reduce the effects of lead on the blood cells, probably through a direct chelating effect or alternatively via blocking the lead mediated reactions which damage the circulating blood cells. Regarding the activity of serum enzymes, which are reliable indicators of tissue damage, this was significantly reduced in rats that had access to quercetin. Probably, this is a direct reflection of the quercetin-induced inhibitory effects on the tissue damaging effects of lead, especially those targeting the liver tissue. The decreased levels of creatinine, urea and BUN express recovery of the renal functions and the success of quercetin to significantly alleviate the lead-induced renal toxic effects. The improvement of the levels of antioxidant markers, including total thiols, catalase and glutathione in rats that had access to quercetin indicate the recovery of the endogenous antioxidant system. On the other hand, the oxidant markers, including H_2_O_2_ and MDA, which express the activity of ROS, were significantly decreased in these rats. This reflects the antioxidant activity of quercetin to inhibit the oxidative reactions and scavenge free radicals. This also implies the improvement of the oxidant–antioxidant balance. Through the aforementioned mechanisms, quercetin can ameliorate the lead-induced oxidative stress and the resultant oxidative damage. The improvement of the hematological and biochemical profiles of the presently lead exposed rats which received quercetin might approve the antioxidant property of quercetin in case of heavy metals toxicity. Quercetin as a dietary supplement presumably acts synergistically with the endogenous antioxidant molecules to alleviate the existing oxidative damage and to sustain the antioxidative status. Counteracting the induced oxidative damage greatly contributes to restore the functional parameters toward the control levels. The present findings might provide evidence that phytoconstituents (phytochemicals), such as flavonoids, can act as efficient antioxidants and confer beneficial effects to mitigate the oxidative stress induced by heavy metal toxicants. However, more investigations at the molecular level are recommended for more clarification of the protective mechanisms through which quercetin can act as a potent antioxidant in case of various toxicities.

## Figures and Tables

**Table 1:** Hematological parameters in rats exposed to lead, and rats exposed to lead and given quercetin phytosome, compared to the untreated control rats.

Parameter	Control	Lead	Lead and Quercetin Phytosome
RBCs count (106/mm^3^)	5.77 ± 0.08	4.17 * ± 0.19	5.34 ** ± 0.07
Total leucocytic	7.11 ± 0.04	5.12 * ± 0.31	6.38 ** ± 0.06
Count			
(103/mm^3^)			
Hemoglobin (Hb)	12.90 ± 0.21	9.64 * ± 0.44	11.89 ** ± 0.53
(g/dL)			
Packed cell volume	47.81 ± 0.32	37.22 * ± 0.79	41.67 ** ± 0.60
(PCV%)			

Values are means ± S.D., N = 20. * Significantly different means from control (*p* ˂ 0.05), ** Significantly different from lead group.

**Table 2:** Biochemical parameters in rats exposed to lead, and rats exposed to lead and given quercetin phytosome, compared to the untreated control rats.

Parameter	Control	Lead	Lead and Quercetin Phytosome
**a.** Levels of total proteins (g/dL), albumin (g/dL), globulin (g/dL), creatinine (mg/dL), urea (mg/dL), BUN (mg/dL) and bilirubin (mg/dL).			
Total proteins	8.33 ± 0.15	5.91 * ± 0.19	7.71 ** ± 0.13
Albumin	3.58 ± 0.06	2.34 * ± 0.18	3.22 ** ± 0.11
Globulin	4.08 ± 0.07	2.57 * ± 0.24	3.19 ± 0.16
Creatinine	0.51 ± 0.14	0.91 * ± 0.47	0.66 ** ± 0.31
Urea	37.66 ± 0.71	63.07 * ± 0.49	46.18 ** ± 0.81
BUN	16.11 ± 1.04	25.35 * ± 1.34	18.83 ** ± 1.62
Bilirubin	7.03 ± 0.31	11.05 * ± 0.46	8.44 ** ± 0.49
**b.** Levels of alanine transferase (ALT) (IU/L), aspartate transferase (AST) (IU/L) and alkaline phosphatase (ALP) (IU/L).			
**ALT**	32.79 ± 0.33	89.17 * ± 0.51	44.29 ** ± 0.64
**AST**	37.88 ± 0.71	83.13 * ± 0.62	46.55 ** ± 0.59
**ALP**	22.87 ± 0.57	77.22 * ± 0.42	35.61 ** ± 0.34
**c.** Levels of total thiols (mmol/L), glutathione (µg/mL), catalase(IU/L), malondialdehyde (MDA) (nmol/mL) and hydrogen peroxide (H_2_O_2_) (mmol/L).			
**Total thiols**	2.46 ± 0.27	0.231 * ± 0.041	1.75 ** ± 0.39
**Glutathione**	42.41 ± 1.41	15.09 * ± 0.37	33.19 ** ± 1.12
**Catalase**	54.11 ± 1.63	29.15 * ± 1.05	45.11 **± 1.28
**MDA**	319.11 ± 3.23	431.17 * ± 3.78	361.27 ** ± 2.62
**H_2_O_2_**	41.37 ± 1.60	86.11 * ± 1.13	55. 27 ** ± 1.18

Values are means ± S.D., N = 20. * Significantly different from control (*p* ˂ 0.05), ** Significantly different from lead group.

## Data Availability

Data supporting the findings in the present work are all available in the article.
